# Optimization of Mordenite Membranes Using Sucrose Precursor for Pervaporation of Water-Ethanol Mixtures

**DOI:** 10.3390/membranes11030160

**Published:** 2021-02-25

**Authors:** Abdulaziz A. Alomair, Yousef Alqaheem

**Affiliations:** Petroleum Research Center, Kuwait Institute for Scientific Research, Safat 13109, Kuwait; yqaheem@kisr.edu.kw

**Keywords:** pervaporation, mordenite, carbon, ethanol

## Abstract

Post-treated mordenite membranes were prepared using sucrose (C_12_H_22_O_11_) as a carbon precursor to block any pinholes and defects in the zeolite layer. The pervaporation (PV) process was used to separate ethanol from the water. The effects of the sucrose concentration and the pyrolysis temperature (650–850 °C) were investigated, and the resulting high separation performance compared to those post/pre-treated membranes was reported in the literature. In this study, mordenite carbon membranes yielded a water/ethanol separation factor of 990.37 at a water flux of 9.10 g/m^2^h. The influence of the operating temperature on the performance of the membrane also was considered. It was concluded that the selective adsorption of water through zeolite pores was achieved. The entire preparation procedure was achieved using a rapid, low-cost preparation process.

## 1. Introduction

The preparation of zeolite membranes and their pre/post-treatment methods have been the subject of intense research in recent years [1,2,3,4,5,6,7,8,9,10,11,12,13]. In our previous studies [10,11], the preparation of different types of zeolite membranes including mordenite membrane using carbon precursors were illustrated and achieved successfully. However, the performance of the prepared mordeinte membrane was lower than those reported in the literature. Therefore, the preparation method was reinvestigated to optimize the overall performance in terms of selectivity and permeability. In this paper, the preparation of highly water-selective mordenite carbon composite membranes for ethanol/water mixtures is illustrated. Due to the hydrophilic characteristic of mordenite, preferential water adsorption should occur, hindering the passage of alcohol through the zeolitic pores [14,15]. Purified ethanol (bioethanol) is considered to be a promising fuel that could be a substitute for gasoline in the future since it is considered to be both a renewable resource and environmentally friendly [16]. The combustion of ethanol releases less greenhouse gases, which is important given that many studies have revealed that 70% of the carbon dioxide in the atmosphere is produced by the combustion of fossil fuels [17,18,19]. Therefore, purified ethanol has attracted considerable interest among many researchers as an alternative to the use of petroleum-based fossil fuels. However, in order to use ethanol as a fuel, the majority of the water must be removed because its presence is undesirable due to the its formation of separating mixtures with hydrocarbons [20,21,22,23,24,25]. To remove the water, conventional distillation generally has been used, but the removal efficiency can only produce a purity of 96% due to the formation of a water-ethanol azeotrope that has a low boiling point [10]. However, PV technology is considered to be a promising separation technique for mixtures that are difficult to separate, especially azeotropes and close-boiling mixtures. In this study, zeolite membranes were used in the PV process to accomplish this task as they have been proven to be beneficial in this effort, due to their uniform microstructure and molecular sieving properties. However, the conventional preparation methods, i.e., the in situ and secondary-growth methods, require a considerable amount of time to fabricate these membranes. Therefore, a similar approach used in our previous studies was considered to reduce time required for the fabrication processes by using a carbon precursor in the processes to form carbon-mordenite composite membranes.

## 2. Experimental Section

**Figure 1:**
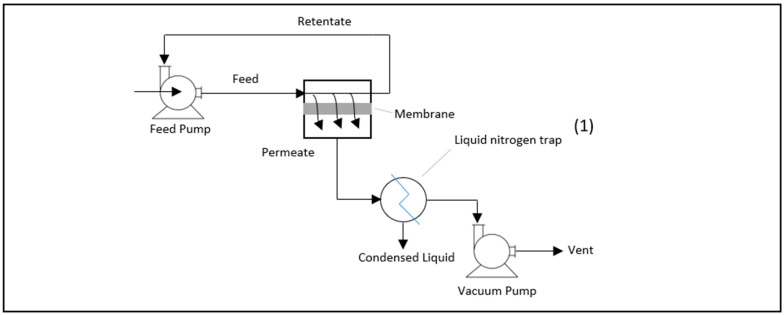
Schematic diagram of the PV unit with membrane module.

Similar to our previous work [10,11], a ready-made mordenite zolite paste was prepared by mixing equal weights of preformed synthetic mordenite (obtained from Eka Nobel, Amsterdam, The Netherlands) and deionized water. Then, a 0.5 g sample of the paste was used to coat the surface of the support disc. The support disc used in this study was a porous 0.5 μm, circular, stainless steel disc that had a thickness of 1.5 mm and a diameter of 25 mm (obtained from Aegis Advanced Materials, Ltd., Bewdley, UK). The coated support was left to dry before the sucrose solution was applied. The sucrose solution was prepared with different sucrose–water combinations, i.e., 1:1, 2:1, and 3:1, by weight. A 0.5 g quantity of the sucrose solution was used to cover the paste under a low vacuum system to provide mild suction through the membrane. Then, a pyrolysis procedure was performed using a tubular furnace with a heating rate of 5 °C/min to reach different temperatures (650–750 °C), which was mainatined for a period of 4 h. The membrane that was produced was attached to a nonporous stainless-steel washer, resulting in a membrane that covered a surface area that had a diameter of 20 mm. The membrane was evaluated using a laboratory-scale PV system (Figure 1) with a feed rate of 130 mL/min and a vacuum pressure of 8 Pa at the permeate side. In the PV process, the feed was in the liquid phase, but the permeate was a vapour due to the pressure difference across the membrane that caused the pressure driving force. For instance, the pressure on the feed side normally is elevated to ensure that there it is different from the vapour pressure of the feed components. Therefore, the pressure on the permeate side should be adjusted so that it is considerably lower than the vapour pressure of the permeate components. Consequently, a vacuum pump was used for this purpose rather than using the sweep gas system to recover the permeate sample. The permeate sample was collected after about 6–7 h by condensing it in a cold trap surrounded with liquid nitrogen. Then, the sample was weighed and analyzed using a gas chromatograph (REX, GC-8810) packed with a thermal conductivity detector (TCD). The performance of the membrane was estimated using the separation factor (α*_i,j_*) (Equation (1)) and the total flux (*F*) (Equation (2)), where (*W_i_*) and (*W_j_*) are the weight compositions of the binary components of the mixture; (*W_P_*) and (*W_F_*) are the weight compositions of the permeate and feed, respectively; (*m_s_*) is the weight of the permeate sample that was collected; (*A*) is the surface area of the membrane; and (Δ*t*) is the duration of the experiment:

(1) $$\alpha_{i,j}=\frac{W_{P,i}\;W_{F,j}}{W_{P,j}\;W_{F,i}}$$

(2) $$F=\frac{m_{s}}{A\;\Delta t}$$

## 3. Results and Discussion

**Figure 2:**
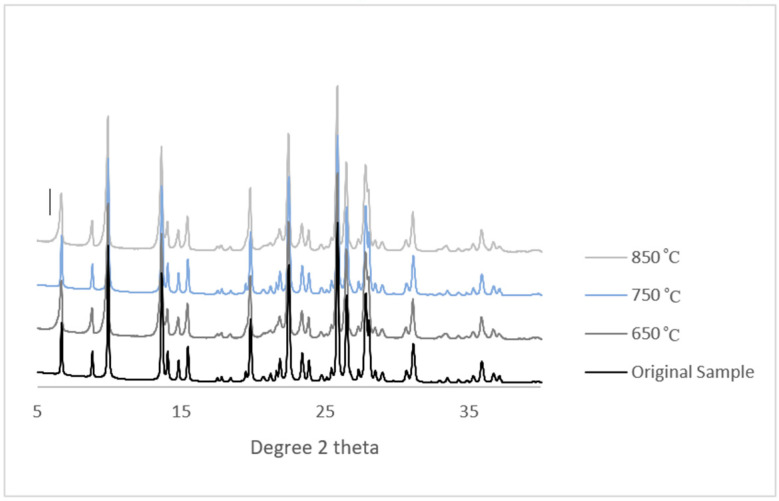
XRD comparison between the mordente at different pyrolysis temperatures.

In this study, mordentite membranes were subjected to different elevated temperatures during the pyrolysis process to carbonize the sucrose precursor. These temperatures were 650 °C, 750 °C and 850 °C. Therefore, the stability of the mordenite was tested at these temperatures, and the results were compared to its original state in Figure 2 using X-ray diffraction (XRD) (obtained from Rigaku Americas Corporation, Austin, TX, USA). The XRD patterns shown in Figure 2 indicated that the crystal structure was unaffected by thermal treatment since the entire pattern had similar positions of the key peaks, which indicated the stability of the mordenite at these temperatures.

**Figure 3:**
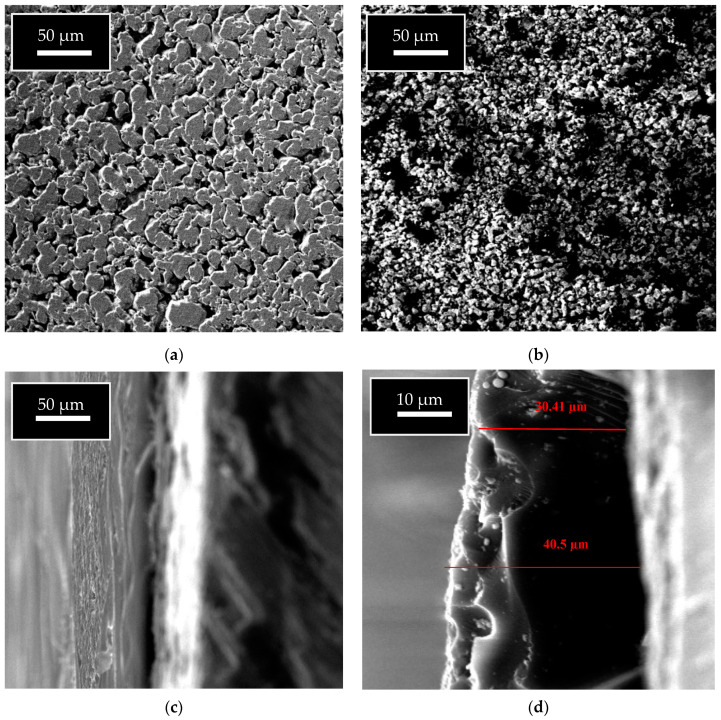
SEM images at 100 µm: (**a**) Stainless steel support; (**b**) carbon-mordenite composite layer (50%) concentrations of sucrose solution; (**c**) cross-section of carbon-mordenite at 50 µm; (**d**) cross-section of carbon-mordenite at 10 µm.

**Figure 4:**
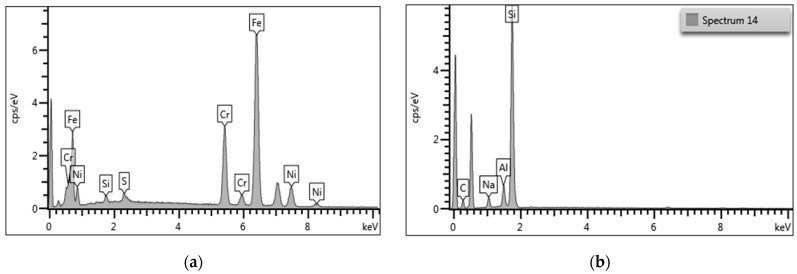
EDX of (**a**) stainless steel support; (**b**) carbon-zeolite composite membrane using 50% concentrations of sucrose solution.

After conducting the pyrolysis with different ratios of sucrose, the excess carbon (which was lightly attached to the surface of the membrane) was removed carefully. A Scanning Electron Microscope (SEM), (JEOL, JSM-IT300, obtained from Rigaku Americas Corporation, Austin, TX, USA) was used to assess the remaining zeolite and carbon on the support disk and to provide detailed images of the surface of the membrane. The SEM images indicated that crystals had been deposited on the surface of the support. Figure 3 shows the comparison between the support disks before and after synthesis. To obtain an additional description, an Energy-dispersive X-ray spectrometer (EDX), obtained from Oxford Instrumentation, was used for the elemental analysis of the surface of the disk before and after the synthesis. Figure 4 shows that the main components on the synthesized membrane were alumina, silica, and carbon, which reflected the abundant coverage of zeolite-carbon across the support. The thickness of the membrane was approximately 35 µm. Also, the mass of the support disk was estimated throughout the synthesis process, as listed in Table 1.

**Figure 5:**
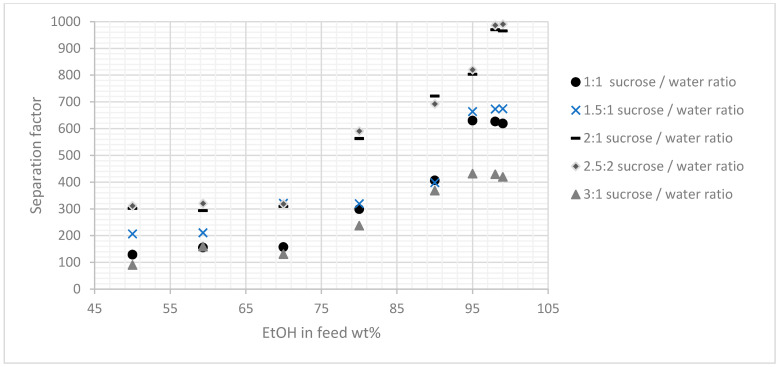
Effect of different concentrations of sucrose on the separation factor (+/−6) at different EtOH feed concentrations.

**Figure 6:**
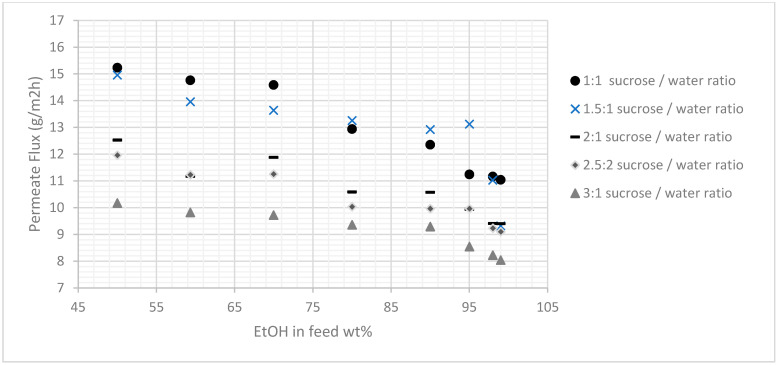
Effect of different sucrose concentrations on Permeate Flux (+/−0.5) at different EtOH feed concentrations.

After the membranes were prepared using the procedure described, various ethanol/water mixtures, i.e., mixtures containing 50, 60, 70, 80, 90, 95, 98, and 99 wt% ethyl alcohol, were evaluated. Figure 5 and Figure 6 present the results, and they indicated that the membranes prepared by this method had been synthesized successfully and that they had high separation factors to those membranes reported in the literature and relatively good fluxes. The effect of the carbon precursor solution also was considered and therefore evaluated at four different concentrations, i.e., sucrose: Water concentrations of 1:1, 2:1, 2.5:1, and 3:1. As shown in Figure 5 and Figure 6, the results indicated that the concentration of the carbon precursor solution had a significant influence on the performance of the membranes in terms of fluxes and separation factors, and to a certain extent, the relationship between the concentration of the carbon precursor solution and selectivity was proportional. In other words, the membranes with higher concentration of the carbon precursor provided better separation and, consequently, lower fluxes. However, the membrane prepared with a very highly concentrated sucrose solution, i.e., (3:1) did not have a performance that was consistent with the performances of the other four membranes, indicating the difficulty of passing the viscous concentrated sucrose solution through the zeolite paste layer and accumulate mainly on the membrane surface that will eventually lower the overall performance.

**Figure 7:**
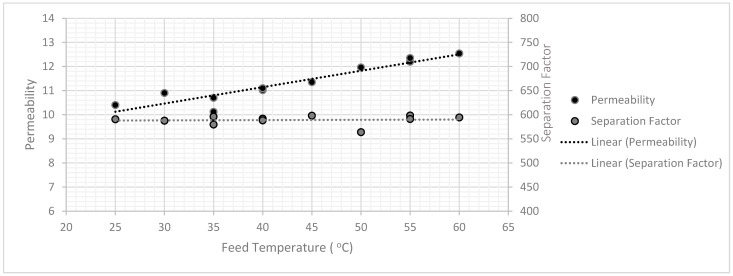
Illustration of temperature effect on permeate fluxes (+/−0.5) and separation factor (+/−6) at the constant feed composition of 50% wt.

Figure 5 indicates that the 2.5:1 sucrose/water ratio yielded a better performance in terms of selectivity and relatively average flux (Figure 6) than the other two concentrations. Therefore, other pyrolysis temperatures were conducted using this concentration for further examination (Table 2 and Table 3). Three pyrolysis temperatures were used in this study i.e., 650, 750, and 850 °C. It was observed that 750 °C was relatively the best pyrolysis condition to produce a selective membrane. This was attributed to the formation of a more suitable structure, since the concentration of the precursor increased as the d-spacing values decreased. The operating temperature also was considered and subjected to the membrane prepared with the pyrolysis condition of 750 °C, where a range of different temperature was used with the feed composition of 80%, as illustrated in Figure 7. The results indicated that the operating temperature influenced the permeate flux slightly without affecting the selectivity. Also, the pervaporation separation index (PSI) was determined using Equation (3):

(3) PSI=J(α−1).

In order to ensure the repeatability of the preparation method, 4 replicates were performed at 25 °C and a feed composition of 80%, and the flux was 10.4 +/− 0.5 g/(m^2^ h). The separation factor was 586 +/− 6. The results of this study were compared to those reported in the literature as shown in Table 4, and the comparison indicated that our membranes were more selective than most of the others, and it also had a much easier and less time consuming procedure that used an inexpensive and renewable precursor, i.e., sucrose.

## 4. Conclusions

The influence of the conditions of the pyrolysis synthesis during the preparation of the mordenite membranes was related to their performance in the dehydration of water/ethanol mixtures by pervaporation. XRD analysis indicated the stability of the mordeinte at the different pyrolysis temperatures used in the study. The optimum synthesis/pyrolysis temperature was 750 °C using a sucrose solution with sucrose: water ratios of 2.5:1. For these synthesis conditions, the membranes yielded water/ethanol separation factors of approximately 990 and water fluxes of 9.10 g/m^2^h. Therefore, those conditions favored the mobility and passage of water through the membrane, leading to maximum fluxes and separation factors. Compared to the literature, the results obtained from this preparation technique led to rapid synthesis, high-selectivity, high reproducibility, and low-cost of the preparation process. Therefore, this work is promising for use in the scale-up fabrication route.

## Figures and Tables

**Table 1:** Weight of the carbon-mordenite membrane during preparation using 50% concentrations of sucrose solution.

Membrane	Weight (Grams)		
	Sample 1	Sample 2	Sample 3
S.S disc	4.206	4.189	4.195
S.S disc + zeolite paste	4.836	4.864	4.706
S.S disc + zeolite paste + sucrose	5.132	5.207	5.198
Membrane after pyrolysis	4.220	4.201	4.211
Mass of carbon plus zeolite	0.014	0.012	0.016

**Table 2:** Effect of different pyrolysis temperatures on the perfromance of the mordenite membrane using 2.5:1 sucrose: water ratio.

Pyrolysis	Feed (EtOH wt%)		Permeate	Separation	PSI
Temperature (°C)	H_2_O	EtOH	Flux (g/m^2^h) (+/−0.5)	Factor (+/−6)	
650	20	80	14.9	81	1195
650	10	90	14.6	125	1821
650	1	99	13.8	201	2770
750	20	80	10.0	590	5917
750	10	90	9.9	692	6889
750	1	99	9.1	990	9005
850	20	80	8.0	498	3996
850	10	90	7.5	654	4934
850	1	99	6.3	834	5288

**Table 3:** Component percentage of feed and permeate using different pyrolysis temperatures with 2.5:1 sucros:water ratio.

Pyrolysis	Feed (g) (wt%)		Feed (mol%)		Permeate (wt%)		Permeate (mol%)	
Temperature (°C)	H_2_O	EtOH	H_2_O	EtOH	H_2_O	EtOH	H_2_O	EtOH
650	20	80	39.02	60.98	95.31	4.69	98.11	1.88
650	10	90	22.14	77.86	93.32	6.68	97.28	2.72
650	1	99	2.52	97.48	67.04	32.96	83.88	6.12
750	20	80	39.02	60.98	99.32	0.67	99.73	0.26
750	10	90	22.14	77.86	98.72	1.28	99.49	0.51
750	1	99	2.52	97.48	90.91	9.088	96.24	3.76
850	20	80	39.02	60.98	99.21	0.79	99.69	0.31
850	10	90	22.14	77.86	98.64	1.36	99.46	0.53
850	1	99	2.52	97.48	89.39	10.61	95.57	4.43

**Table 4:** Literature survey on ethanol-water separation using moredite membrane.

Preparation Method	Support	Feed Temperature (°C)	Separation Factor	Flux (g/m^2^h)	Reference
Carbon pretreatment	stainless-steel	25	990	9.1	Current study
Hydrothermal synthesis	α-Al_2_O_3_	25	150	200	[4]
Seeded hydrothermal synthesis	α-Al_2_O_3_	25	60	5.3	[5]
Post-treatment with hydrochloric acid solution	α-Al_2_O_3_	25	368	100	[6]
Post-treatment with oxalic acid	α-Al_2_O_3_	75	>10,000	53	[9]
Post-treatment in the hydrochloric acid solution	α-Al_2_O_3_	50	>10,000	60	[11]
Alkaline post- treatment	α-Al_2_O_3_	150	139	160	[22]
Alkaline post- treatment	α-Al_2_O_3_	150	900	260	[23]
microwaveassisted synthesis	α-Al_2_O_3_	75	7500	1100	[25]
